# The Modern Presentation, Etiology, and Rate of Occurrence of Cushing Syndrome Differs when Compared to Prior Population-Based Studies

**DOI:** 10.1016/j.aed.2025.11.010

**Published:** 2025-11-27

**Authors:** Thomas Matoska, James W. Findling, Tracy S. Wang, Douglas B. Evans, Sophie Y. Dream, Bradley R. Javorsky, Nathan T. Zwagerman, Ty B. Carroll

**Affiliations:** 1 Department of Radiation Oncology, Medical College of Wisconsin, Milwaukee, Wisconsin; 2 Division of Endocrinology and Molecular Medicine, Department of Medicine, Medical College of Wisconsin, Milwaukee, Wisconsin; 3 Division of Surgical Oncology, Department of Surgery, Medical College of Wisconsin, Milwaukee, Wisconsin; 4 Division of Breast and Endocrine Surgery, Department of Surgery, University of Alabama at Birmingham, Birmingham, Alabama; 5 Department of Neurological Surgery, Medical College of Wisconsin, Milwaukee, Wisconsin; 6 Division of Endocrinology, Diabetes, and Metabolism, Department of Medicine, University of Wisconsin-Madison School of Medicine and Public Health, Madison, Wisconsin

**Keywords:** Cushing syndrome, clinical presentation, Cushingoid, ACTH-independent

## Abstract

**Background/Objective:**

Endogenous Cushing Syndrome (CS) is considered a rare disorder, with European population studies estimating an incidence of 1.5-3.2 cases/million population/year and Cushing disease (CD) being recognized as the most common etiology. The purpose of this study is to characterize the etiologies of endogenous CS, CS severity, and case frequency of CS at a single institution.

**Methods:**

Between May 2017 and December 2022, clinic records from a single tertiary care medical center were reviewed to identify Wisconsin residents who were newly diagnosed and treated for CS. We collected information on the etiology, biochemical data, and evidence of physical features of CS.

**Results:**

A total of 185 patients diagnosed with and treated for endogenous CS in our state. Of these, 111 (60%) had adrenal CS, 68 (36.8%) had CD, and 6 (3.2%) had ectopic ACTH syndrome. Patients with adrenal CS had less severe hypercortisolism compared to CD and ectopic ACTH syndrome and a lower frequency of Cushingoid appearance at diagnosis. The case frequency within our institution’s state was 7.2 cases/million population/year (total population). Adjusting for institutional state-wide market share, we estimate a more accurate state-wide case frequency of CS to be 11.1 cases/million population/year.

**Conclusion:**

Adrenal CS was more common than CD in our cohort and often presented with milder cortisol excess that still required treatment. The relative case frequency of endogenous CS in Wisconsin may approach 11 case/million population/year based on this single-institutional data, but further population-based studies are needed to estimate the incidence of endogenous CS.

**Highlights**

• Cushing disease has historically been reported to be the most common cause of neoplastic hypercortisolism; however, our data show that adrenal Cushing syndrome (CS) accounts for 60% of cases of neoplastic hypercortisolism
• Many patients with clinically significant do not present with classic physical features of CS
• CS is likely much more common than most prior reports suggest

**Clinical Relevance**

Cushing syndrome (CS) is often considered a rare disorder, but modern studies of its incidence are lacking. The current single center study suggests that CS is more common than previously described and highlights that adrenal CS is the most common etiology. These data should increase recognition and screening for this potentially life-threatening disorder.

## Introduction

Since Dr Harvey Cushing’s first report of clinical hypercortisolism in 1912,^1^ this well-known endocrinopathy has been considered to be rare. Dr Cushing correctly speculated that basophilic pituitary adenomas were the cause of adrenal hyperactivity in his initial monograph and, appropriately, this disorder is recognized as Cushing disease (CD).^2^ The manifestations of endogenous or exogenous excessive glucocorticoid exposure [Cushing syndrome (CS)] include facial fullness, increased dorsocervical fat accumulation, truncal obesity with wide, violaceous striae, cutaneous wasting, and muscle weakness-findings that are evident to even the most inexperienced clinician.^3^ In the past 10 to 15 years, there has been increasing recognition of milder degrees of neoplastic cortisol excess that are associated with increased morbidity and mortality. Many of these patients, particularly those with adrenal dependent hypercortisolism, lack many of the overt physical features of cortisol excess, suggesting that the classic phenotype is a caricature of neoplastic hypercortisolism. Because many clinicians do not consider CS unless patients present with an overt phenotype, the diagnosis is often delayed or missed entirely.^4^

Historically, studies have shown that CD is the most common cause of neoplastic hypercortisolism.^5^, ^6^, ^7^, ^8^, ^9^ The widespread use of abdominal cross-sectional imaging has led to increased diagnosis of adrenal nodules.^10^ Since 20% to 40% of adrenal nodules may be associated with mild autonomous cortisol hypersecretion (MACS),^11^ the recognition and prevalence of adrenal CS may be increasing as well. Perhaps due to lack of screening, endogenous CS is referred to as a rare disease. Several population-based studies from outside the United States (US) that use national healthcare databases have estimated an incidence for all types of neoplastic CS at 1.5-3.2 cases/million population/year (Table 1).^5^^,^^7^^,^^12^, ^13^, ^14^, ^15^, ^16^ One US study used a commercial insurance database to estimate the incidence of neoplastic CS to be 39.5 to 48.6 cases/million population/year.^17^ Another recent study demonstrated abnormal dexamethasone suppression testing in nearly 25% of patients with difficult to control diabetes but did not confirm neoplastic CS in these individuals.^18^ The results of these studies suggest that neoplastic CS may be much more common than previously reported, especially in the modern era of less significant hypercortisolism that still is worthy of treatment.

**Table 1:** Studies of Cushing Syndrome Incidence

Author (publication date)	Data collection time period	Study location	Patient identification	Incidence (cases/million population/y)^a^
Etxabe (1994)	1975-1992	Spain	Medical Records	2.4
Lindholm (2001)	1985-1995	Denmark	National Database	1.2-1.7
Arnardottir (2011)	1955-2009	Iceland	National Database	1.5
Bolland (2011)	1960-2005	New Zealand	Medical Records	1.8
Broder (2015)	2007-2010	USA	Commercial Database	39.5-48.6
Wengander (2019)	2002-2017	Sweden	National Database	3.2
Ragnarsson (2019)	1987-2013	Sweden	National Database	1.6^b^
Ahn (2021)	200-2017	S Korea	National Insurance Database	1.5^c^
Matoska (2025)^a^	2017-2022	WI, USA	Medical Records	7.2-11.4

a Incidence in all studies other than Matoska which calculated case frequency at a single institution.

b Cushing disease only.

c Adrenal dependent Cushing syndrome only.

Our clinical experience suggests that adrenal dependent CS is more common than CD at our institution, and that these patients may have less severe features and laboratory measurements of hypercortisolism. We acknowledge that with a single-institutional study that incidence cannot be measured, however, we seek to calculate the relative case frequency of endogenous CS in the context of our state’s population. This study reports the CS presentation and degree of cortisol excess among subtypes of CS in Wisconsin along with estimating relative case frequency in the state by evaluating market share data and patients who were diagnosed and treated at a single tertiary care medical center.

## Methods

### Patient Cohort

After Institutional Review Board approval (protocol number: PRO00046343), endocrinology, neurosurgery, and endocrine surgery clinic records from May 1, 2017 to December 31, 2022 (5.67 years) were reviewed. Patients who were newly diagnosed with and treated for neoplastic CS at Froedtert and the Medical College of Wisconsin were identified. Our center has both well-recognized tertiary care pituitary neurosurgery and endocrine surgery programs that have a wide regional referral base including eastern and central Wisconsin as well as northern Illinois. Initial testing for CS was initiated for a variety of indications including metabolic abnormalities (eg diabetes, hypertension, and osteoporosis), incidental adrenal nodules, pituitary imaging abnormalities, and/or classic physical features of CS. The diagnosis of CS was established with guideline supported biochemical testing and appropriate imaging. All patients had confirmation of abnormal screening biochemical testing and additional differential diagnostic testing completed. To be included in the study population, patients had to have an initial diagnosis of CS and receive treatment (surgery or cortisol directed medical therapy) at our institution. Only patients with a Wisconsin mailing address at time of diagnosis were included in the patient cohort. Patients with recurrent CS, patients who did not receive treatment for neoplastic CS, patients diagnosed outside of the specified study period, patients not residing in Wisconsin, and those with exogenous or non-neoplastic CS patients were excluded.

Classic features of hypercortisolism in our population were assessed qualitatively based on the initial examination at our center by one of our endocrine clinicians. Specific physical features that were consistent with neoplastic hypercortisolism (Cushingoid appearance) included the presence of facial rounding/plethora, truncal obesity with increased dorsocervical and supraclavicular fat accumulation, cutaneous wasting, violaceous abdominal striae, hirsutism, and myopathy.

### CS Subtype and Severity Analysis

Data collected for each patient included etiology of neoplastic CS, basal ACTH levels at diagnosis, sex, type of treatment received, and presence of physical features of CS at diagnosis. Descriptive statistics were calculated using Chi-square and ANOVA tests. These tests were employed to evaluate differences in biochemical testing values, demographics, and classic physical features for each etiology of CS. Linear regression modeling was also used to test these relationships both with and without adjustments for late-night salivary cortisol (LNSC), 1 mg overnight dexamethasone suppression test (DST), and sex.

#### Relative Case Frequency Calculation

Relative case frequency for our patient cohort was defined as the number of patients residing in Wisconsin who were diagnosed with and treated for at our institution, relative to the Wisconsin population. Population data was extracted from the United States Census Bureau: Wisconsin 2020 Census.^19^ To better represent the population at risk, the denominator was defined as the sum of the Wisconsin county populations representative of patients’ residential zip codes. Wisconsin counties that were not represented by the patient cohort were excluded from the population at risk. The relative case frequency of CS was calculated using the following equation:

$$\frac{\text{Number}\;\text{of}\;\text{patients}\;\text{diagnosed}\;\text{with}\;\text{Cushing}\;\text{syndrome}\;\text{May}\;2017\;\text{and}\;\text{December}\;2022}{Population\;at\;risk\;\left(sum\;of\;WI\;county\;populations\right)/million\;population/5.67years}$$

Market share data of our institution, specifically hospital discharges with primary CS related ICD-10 codes (E240, E243, E249), was then obtained to estimate a more accurate (adjusted) relative case frequency of CS in Wisconsin, as there are other medical centers that also treated CS within this region.

## Results

### Diagnosis

During the study period, 185 patients met the biochemical criteria for the initial diagnosis of neoplastic CS and received treatment at Froedtert and the Medical College of Wisconsin. Adrenal CS accounted for 111 (60%) of all patients diagnosed and treated. CD represented 68 (37%) patients, and 6 (3%) patients had ectopic ACTH syndrome (EAS). Of the total group, 135 (73%) were female. The mean age at diagnosis for all etiologies of neoplastic CS was 52.4 years (SD ± 14.7 years). Regarding treatment, 173 (94%) patients received a surgical procedure (pituitary adenomectomy or adrenalectomy) to treat their CS, and 12 (6%) patients received medical therapy alone.

At diagnosis, 117 (53%) patients had classic physical features of CS on physical exam. Considering the etiology of CS, 49 of 111 (44%) patients with adrenal CS, 62 of 68 (91%) patients with CD, and all 6 patients with EAS had classic physical features of CS at diagnosis (Table 2).

**Table 2:** Summary of Demographical Patient Information by CS Subtype

Patient characteristic	All subtypes	Adrenal	CD	EAS	*P*-value
Number of patients	185	111	68	6	
Mean age at dx (SD)	52.4 (14.7)	56.0 (13.4)	45.7 (14.4)	61.9 (13.2)	<0.0001
Female sex (vs male)	135 (73%)	81 (73%)	51 (75%)	3 (50%)	0.42
Classic physical features at diagnosis	117 (63%)	49 (44%)	62 (91%)	6 (100%)	<0.001

Abbreviations: CD = Cushing syndrome, EAS = ectopic ACTH syndrome, SD = standard deviation.

### CS Severity Analysis

Table 3 summarizes biochemical testing data for the entire cohort and for each CS etiology. Three primary diagnostic tests for CS (DST, LNSC, and urine free cortisol [UFC]), were abnormal in 97.6%, 75.1%, and 58.0% of patients, respectively. Patients with EAS had much higher LNSC, DST, UFC, and plasma ACTH than the other etiologies. Patients with CD had higher LNSC, DST, UFC, and plasma ACTH compared to adrenal CS. Patients with adrenal CS had lower plasma ACTH than the ACTH-dependent etiologies.

**Table 3:** Summary of Biochemical Testing Each CS Subtype

Biochemical test	All CS	Adrenal	CD	EAS	*P*-value
LNSC [μg/dL]	0.419 (0.641)	0.239 (0.366)	0.467 (0.402)	3.178 (0.928)	<0.001
DST [μg/dL]	9.4 (12.0)	6.5 (8.7)	11.7 (7.5)	42.5 (40.5)	<0.001
UFC [μg/24 h]	158.7 (408.5)	64.2 (92.8)	151.1 (287.4)	1514.2 (1278.9)	<0.001
Basal ACTH [pg/mL]	32.4 (46.8)	6.3 (5.1)	62.1 (31.2)	180.05 (116.3)	<0.001

Abbreviations: CS = Cushing syndrome, CD = Cushing disease, EAS = ectopic ACTH syndrome, LNSC = late night salivary cortisol (normal <0.116 μg/dL), DST = post 1 mg dexamethasone suppression cortisol (normal ≤1.8 μg/dL), UFC = urinary free cortisol (normal <45 μg/24 h).

Mean values (standard deviation).

When comparing the 117 patients (63% of CS cohort) with classic physical features to those without classic physical features, there were differences in biochemical testing. The mean LNSC, DST, and UFC were all higher in those patients with classic physical features (Table 4). With the use of logistics regression modeling, classic physical features were found to be more likely with higher LNSC, odds ratio of 1.06 (95% CI, 1.02-1.11, *P*-value <0.05) and higher DST, odds ratio 1.10 (95% CI, 1.05-1.17, *P*-value <0.001). Additional modeling showed that after controlling for LNSC, DST, and sex, DST (odds ratio 1.08, 95% CI, 1.02-1.15, *P*-value <0.05) and sex (odds ratio 0.40 (95% CI, 0.18-0.88, *P*-value <0.05) were independently correlated with development of classic physical features.

**Table 4:** Biochemical Data With and Without Classic Physical Features of Hypercortisolism

Biochemical & patient characteristics	Total #	CS features Present	CS features Absent	*P*-value
LNSC [μg/dL]	165	0.520 (0.722)	0.237 (0.410)	<0.001
DST [μg/dL]	169	11.9 (13.3)	5.7 (8.7)	<0.001
Mean UFC [μg/24 h]	131	197.6 (478.1)	41.9 (104.7)	<0.001
Female	135	92 (68%)	43 (63%)	<0.05
Male	50	25 (50%)	25 (50%)	

Abbreviations: CS = Cushing syndrome, LNSC = Late night salivary cortisol (normal <0.116 μg/dL), DST = post 1 mg dexamethasone suppression cortisol (normal ≤1.8 μg/dL), UFC=Urinary free cortisol (normal <45 μg/24 h).

Mean values (standard deviation/percentage).

#### Relative Case Frequency of Neoplastic CS

This cohort identified patients that resided in 27 of Wisconsin’s 72 counties. These 27 counties had a combined 2020 census population of 4 512 001 (76.6% of total state population). As 185 cases of CS were diagnosed and treated, this represented a relative case frequency for all etiologies of neoplastic CS of 7.2 cases/million population/year at our institution relative to the counties represented by this population (Fig.). Our institution is a high-volume center for CS care, but data from the Wisconsin Hospital Association indicated that, on average from the 2019-2023, our institution accounted for 48.6% of Wisconsin’s total number of hospital discharges where the primary diagnosis was related to CS. Using this information, a market share-adjusted relative case frequency of 11.4 cases/million population/y was estimated with the following equation:

$$\frac{185\;cases}{5.89\;million\;population\;x\;5.67\;years\;x\;48.6\%}$$

**Figure:**
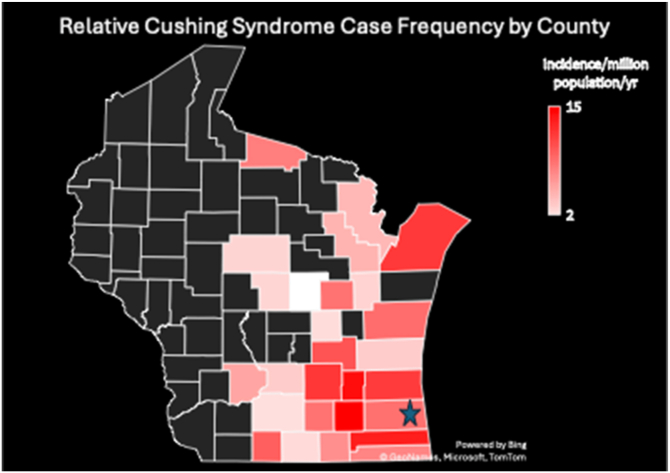
A heat map of Wisconsin and the counties represented by the study cohort. Red indicates a higher relative case frequency in that county. = Location of study *Center*.

Considering each etiology of CS separately, we found adrenal dependent hypercortisolism to have a relative case frequency and market share-adjusted relative case frequency of 4.3 and 6.8 cases/million population/y, respectively. The unadjusted and adjusted relative case frequency of CD was 2.7 and 4.2 cases/million population/year (Table 5), respectively.

**Table 5:** Case Frequency of Cushing Syndrome by Etiology

Etiology	Number of cases	Case frequency^a^	Adjusted case frequency^b^
All types	185	7.2	11.4
Cushing disease	68	2.7	4.2
Adrenal CS	111	4.3	6.8

Abbreviation: CS = Cushing syndrome.

a Case frequency in cases per million population per year.

b Case frequency in cases per million population per year adjusted for market share.

## Discussion

For over 100 years, neoplastic CS has been considered a rare endocrine disorder. This is supported by multiple international studies using national medical record databases^4^^,^^5^^,^^12^, ^13^, ^14^, ^15^^,^^20^ although a US study, using an insurance database, suggested a much higher incidence.^17^ Historically, CD has been more common than adrenal CS. However, our institutional experience suggests that adrenal CS requiring treatment may be more common than CD. Patients with adrenal CS in our cohort had lower average biochemical testing results and less frequency of Cushingoid features at diagnosis, suggesting that, in the modern era, less severe presentations of clinically significant hypercortisolism are being detected and treated.

The recognition of milder forms of cortisol excess likely plays a role in the increased number of adrenal CS patients at our institution. The observation that MACS exists in 20% to 40% of patients with adrenal nodular disease highlights this observation. Expert consensus guidelines from the European Society of Endocrinology recommending that a solitary abnormal DST constitutes neoplastic hypercortisolism worthy of treatment consideration, may also contribute to the increased diagnosis of neoplastic hypercortisolism.^21^ Even after excluding patients with adrenal CS who lacked classic physical features, we found an estimated case frequency and market-adjusted case frequency of 4.8 and 7.6 cases/million population/year, respectively.

Accordingly, in contrast to every previous publication, this single-institutional study provides evidence that adrenal CS may be than all other etiologies of neoplastic hypercortisolism. Although some of the adrenal CS patients certainly met the diagnostic criteria for MACS, the mean ACTH level was below the reference range and the mean UFC was above the reference range in patients with adrenal CS.

It is not surprising that the physical manifestations of CS in our patients differed depending on the magnitude of the hypercortisolism: only 44% of patients with adrenal CS exhibited overt CS features in contrast to 91% of patients with CD and all the ectopic patients with ACTH. The “physical features of CS” were reviewed from the first patient encounter in our clinics which may have introduced bias as most of the initial visits were accompanied by some abnormal laboratory studies of HPA axis function. Clearly, when the initial endocrine encounter is accompanied by elevations of LNSC, UFC, and DST, there may be bias to perceive the patient as appearing more “Cushingoid.” Nonetheless, the recognition that even MACS is associated with significant metabolic, skeletal, neuropsychiatric, and cardiovascular morbidity underscores the importance of the diagnosis of all types of neoplastic hypercortisolism. Recently, the CATALYST trial found a prevalence of hypercortisolism of 24% in patients with difficult to control diabetes.^18^ This finding supports the idea that patients may not appear “Cushingoid” but still have clinically significant hypercortisolism. These findings suggest that the threshold for hypercortisolism screening needs to be reevaluated to prevent missing patients with unrecognized hypercortisolism.

Given the single-institutional nature of our study, incidence calculation is inappropriate. Therefore, we sought to estimate the case frequency of endogenous CS relative to our state’s population to obtain an of how frequently the population our institution serves develops CS. Our study identified 185 newly diagnosed and treated patients with CS residing in 27 of the 72 Wisconsin counties during a 5.67-year period. During the course of clinical care, the authors confirmed the diagnosis of neoplastic hypercortisolism in all cases using biochemical and, in most cases, pathologic specimens. This resulted in an estimated relative case frequency of 7.2 cases/million population/year. The authors recognize that this case frequency is an underestimation in our state, as there are many other institutions in our area that treat endogenous CS. Thus, market share data was used to more accurately estimate the case frequency of CS in our state. The use of market share data showed that our institution sees just below 50% of all the patients discharged from Wisconsin hospitals with the diagnosis of CS. Using this data, the market-share adjusted case frequency estimate is around 11.4 cases/million population/year. Our study may also underestimate the case frequency of CS at our institution as the latter half of the study inclusion period overlapped with the COVID-19 pandemic, which likely led to compromised or limited health care for many individuals. Thus, based off our limited single institution analysis, there appears to be more endogenous CS diagnoses at our institution in the modern era, but population-based incidence studies are required to make this determination.

It is important to consider why our case frequency calculations are higher than older population-based incidence studies (Table 1). It seems very unlikely that CS is simply more common in Wisconsin than anywhere else. Older studies that reported lower incidences relied on diagnostic coding for their inclusion criteria, which may omit patients that were incorrectly coded, as there are limited ICD codes for hypercortisolism.^5^^,^^12^^,^^15^^,^^16^ Other possible explanations for our higher case frequency of neoplastic CS include the widespread use of more sensitive tests such as late-night salivary cortisol and dexamethasone suppression accompanied by new diagnostic thresholds for the diagnosis. In many studies, UFC was used as a screening test, but it is now appreciated that this test has such poor sensitivity that many experts have suggested abandoning UFC in the screening process (21). Finally, if modern population-based incidence studies were performed, the incidence may be higher due to increased awareness and diagnosis of MACS, which can cause less severe but clinically significant hypercortisolism requiring treatments.

When thinking about how common CS is in the modern era, we should look at the incidence published by Broder et al[17]. The patient selection process from this study did not confirm each case individually by the investigators. While our study could not calculate incidence, we included only those patients who were diagnosed and treated at our institution, limiting the patients included; however, the diagnosis was personally confirmed by the investigators in each case. Conversely, Broder et al used a commercial insurance database that utilized ICD-9 codes to identify patients with CS. As ICD-9 codes lack specificity for the etiology of CS and even included exogenous CS, the investigators were not able to personally verify the diagnosis in each patient. The use of an insurance database may also lead to overrepresentation of certain patient groups or demographics who had better access to care and testing.

There are several limitations of our study. The first being its retrospective nature. It is possible that some patients with CS were missed from the endocrine, neurosurgery, or endocrine surgery clinics. Patients did not all complete a similar diagnostic evaluation; for example, not all patients had UFC performed. Additionally, our results and ability to characterize the commonality of CS in our region is extremely limited due to single-institutional nature of the study. It is likely that many individuals with CS in the 27 counties from which we diagnosed patients had care in other health care systems such as other large, tertiary university systems or other nearby metropolitan centers that provide care to the same geographic area. Accordingly, we acknowledge that our case frequency calculation should not be interpreted as the incidence in our state, rather, a hypothesis generating number that encourages true population-based studies. Another potential limitation impacting the subtyping of CS might be the high volume of adrenal surgery performed at our institution. This may lead to referral bias causing higher numbers of adrenal CS compared to CD. However, our center also does over 40% of the pituitary tumor surgeries in Wisconsin, making our study likely reflective of the population with a balanced and well characterized group of patients with neoplastic hypercortisolism.

There are also several strengths of our study. To our knowledge, this is one of the first studies to suggest that adrenal CS may be more common than previously thought. Additionally, our study emphasizes an important point in the modern era of more sensitive testing and MACS: clinically significant hypercortisolism warranting treatment presents on a vast scale. Additionally, every endogenous CS patient included in the study was seen in our clinics and diagnosed with disease significant enough to receive treatment by a small number of physicians. Endogenous CS diagnosis was then able to be validated by our endocrinology team during the data processing of the project to ensure the number of patients diagnosed at our institution was accurate. While our study cannot estimate the incidence of CS, calculating the relative case frequency at our institution relative to our state’s population has seldom been done before, especially in the United States. We hope that our study will encourage other institutions or health care organizations to obtain their own CS databases and collaboration can ensue to estimate incidence in the future.

The recognition of the importance of even milder degrees of cortisol excess on cardiometabolic morbidity and the appreciation of the high proportion of MACS in patients with adrenal nodular disease has generated more screening for CS. This study provides suggest evidence that adrenal CS may be more common than CD, and a large portion of these patients have clinically (Cushingoid features) and objectively (lower average biochemical testing) less severe hypercortisolism. Additionally, we performed a relative case frequency calculation to identify the frequency of CS diagnoses made at our institution as a function of our state’s population. This calculation showed CS may be more common in the modern era. The results of this study should motivate population-based incidence studies in the United States to better estimate how common CS is today. Eventually, research and dissemination of information regarding CS in the modern era to primary care providers and other non-endocrinologists may lead to increased detection of clinically significant hypercortisolism and improved patient outcomes.

## Conflict of Interest

Dr Carroll is an Editorial Board Member of this journal and was not involved in the editorial review or the decision to publish this article. Findling serves on Advisory Boards for: Corcept Therapeutics, Crinetics, and Diurnal and is an investigator for Recordati. Javorsky receives research support from Chiesi Farmaceutici. Matoska, Wang, Evans, Dream, and Zwagerman have nothing to disclose.

## Funding Sources

None.

## Data Availability

All datasets generated during and/or analyzed during this current study are not publicly available but are available from the corresponding author on reasonable request.
